# Machine learning‐based models to predict the need for neurosurgical intervention after moderate traumatic brain injury

**DOI:** 10.1002/hsr2.1666

**Published:** 2023-10-29

**Authors:** Adrina Habibzadeh, Sepehr Khademolhosseini, Amin Kouhpayeh, Amin Niakan, Mohammad Ali Asadi, Hadis Ghasemi, Reza Tabrizi, Reza Taheri, Hossein Ali Khalili

**Affiliations:** ^1^ Student Research Committee Fasa University of Medical Sciences Fasa Iran; ^2^ USERN Office Fasa University of Medical Sciences Fasa Iran; ^3^ Shiraz Trauma Research Center Shiraz Iran; ^4^ Department of Pharmacology Fasa University of Medical Sciences Fasa Iran; ^5^ Shiraz Neurosurgery Department Shiraz University of Medical Sciences Shiraz Iran; ^6^ Department of Computer Engineering, Shiraz Branch Islamic Azad University, Shiraz University Shiraz Iran; ^7^ Biology and Medicine Faculty Taras Shevchenko National University of Kyiv Kyiv Ukraine; ^8^ Noncommunicable Diseases Research Center Fasa University of Medical Sciences Fasa Iran; ^9^ Clinical Research Development Unit, Valiasr Hospital Fasa University of Medical Sciences Fasa Iran; ^10^ Shiraz Neuroscience Research Center Shiraz University of Medical Sciences Shiraz Iran

**Keywords:** CT scan, machine learning, neurosurgical intervention, prediction, traumatic brain injury

## Abstract

**Background and Aims:**

Traumatic brain injury (TBI) is a widespread global health issue with significant economic consequences. However, no existing model exists to predict the need for neurosurgical intervention in moderate TBI patients with positive initial computed tomography scans. This study determines the efficacy of machine learning (ML)‐based models in predicting the need for neurosurgical intervention.

**Methods:**

This is a retrospective study of patients admitted to the neuro‐intensive care unit of Emtiaz Hospital, Shiraz, Iran, between January 2018 and December 2020. The most clinically important variables from patients that met our inclusion and exclusion criteria were collected and used as predictors. We developed models using multilayer perceptron, random forest, support vector machines (SVM), and logistic regression. To evaluate the models, their F1‐score, sensitivity, specificity, and accuracy were assessed using a fourfold cross‐validation method.

**Results:**

Based on predictive models, SVM showed the highest performance in predicting the need for neurosurgical intervention, with an F1‐score of 0.83, an area under curve of 0.93, sensitivity of 0.82, specificity of 0.84, a positive predictive value of 0.83, and a negative predictive value of 0.83.

**Conclusion:**

The use of ML‐based models as decision‐making tools can be effective in predicting with high accuracy whether neurosurgery will be necessary after moderate TBIs. These models may ultimately be used as decision‐support tools to evaluate early intervention in TBI patients.

## 1 INTRODUCTION

Traumatic brain injury (TBI) is a leading cause of mortality and disability, presenting a significant global public health challenge. Often described as the silent epidemic of our era, TBI carries substantial socioeconomic implications.^1^ Each year, approximately 69 million individuals worldwide experience TBI, with the Southeast Asian and Western Pacific regions carrying the highest burden of cases.^2^ Initial evaluation of TBI involves evaluating the patient's Glasgow coma scale (GCS) score as well as performing a brain computed tomography (CT). According to the GCS, TBIs are classified into mild (13−15), moderate (9−12), and severe (8 or below), depending on their neurological impairment and clinical manifestations.

Although there are guidelines for managing mild and severe TBI, there are few specific guidelines for “moderate TBI.” This disparity is evident when comparing the literature on mild and severe TBI, which far outnumbers research on moderate head injuries. However, it is crucial to recognize that moderate TBI patients who initially appear conscious and able to communicate may later decline and suffer serious consequences.^3^

CT scans play a vital role in the initial assessment of TBI by providing valuable information about intracranial injuries. Recent improvements in CT imaging techniques, such as advanced imaging techniques, have made it easier to spot changes in the structure of the brain, such as white matter abnormalities, diffuse axonal injury, and subtle contusions.^4^, ^5^ Detecting these changes early can aid in predicting future events and guide management decisions.

One significant concern in TBI management is progressive hemorrhagic injury (PHI), which refers to delayed injuries observed on follow‐up CT scans. In the early 1990s, Stein et al.^6^ first described PHI when they observed that almost 50% of head injury patients presented delayed injuries on follow‐up CT scans. Since that groundbreaking discovery, numerous studies have been undertaken to explore and understand PHI further. PHI occurrence is associated with a fivefold increase in clinical deterioration risk, resulting in significant morbidity and mortality in TBI patients.^7^ Therefore, early and accurate prediction of PHI would greatly benefit the assessment of TBI patients, leading to reduced morbidity and mortality.

Currently, routine follow‐up CT scans are performed for TBI patients to evaluate the progression of intracranial injuries after initial CT scans and the need for neurosurgical intervention.^8^, ^9^ However, the indication for routine follow‐up head CT scans in TBI patients is unclear, and convincing evidence is lacking. Repeated CT scanning can result in patients being exposed to radiation unnecessarily. Besides, it may increase the cost of both the healthcare system and patients and increase the workloads of medical and paramedical staff.^10^ Other downsides of unnecessary test ordering are overwhelming the machine, delaying tests for other patients, and the risk of transportation, especially in low‐source settings.

Although the missing progression of a brain injury can cause morbidity and mortality, over the past several years, some studies have been conducted to evaluate the efficacy of routine follow‐up CT scans of the head in patients with mild to moderate TBI and to determine if these scans led to treatment changes.^11^, ^12^, ^13^, ^14^ Several studies recommended observation without routine follow‐up CT scans except in the setting of risk factors such as a fall in GCS or other neurological deteriorations.^15^

Nevertheless, determining which TBI patients would benefit from a follow‐up CT scan or early intervention poses significant challenges. To develop innovative clinical tools using vast data sets and advanced computational resources, machine learning (ML) provides a promising solution. In recent times, numerous clinical diagnostic tools have been developed using ML methods.^16^, ^17^, ^18^

Furthermore, the prediction of patients who may require neurosurgery allows the trauma team to anticipate and allocate necessary resources more effectively. As a result, we aim to develop the first ML‐based models that predict the need for neurosurgical intervention in moderate TBI patients based on data collected within the first hours of a patient's admission.

## 2 METHODS

### 2.1 Study design

This is a retrospective study that is conducted to determine whether an application of ML‐based models could be used to predict the need for neurosurgical intervention in moderate TBI patients with positive initial CT scans. All patients admitted to the neuro‐intensive care unit (ICU) of Emtiaz Hospital, Shiraz, Iran, between January 2018 and December 2020, who met our inclusion and exclusion criteria were included in this study.

We primarily used clinically relevant variables that are routinely collected in the assessment of TBI patients. These variables have established clinical significance in predicting the need for neurosurgical intervention. The most clinically important variables, including age, gender, motor GCS 0, motor GCS 6, Marshall 0, Marshall 6, Hematoma 0, Hematoma 6, Midline shift 0, Midline shift 6, temporal lesions, and bifrontal contusions, were collected and used as predictors. a head CT was considered positive if there was suspicion or a clear indication of traumatic pathology. Marshall CT score is presented in Table 1. It is important to note that 0 represents initial or at admission and 6 is 6 h later.

**Table 1:** Marshall classification score.

Scale	CT findings
Category I	No visible intracranial pathology
Category II	Midline shift of 0−5, basal cistern remains visible, no high or mixed density lesions >25 cm^3^
Category III	Midline shift of 0−5, basal cistern compressed or absent, no high or mixed density lesions >25 cm^3^
Category IV	Midline shift >5 mm, no high or mixed density lesions >25 cm^3^
Category V	Any lesions evacuated surgically
Category VI	High or mixed density lesions >25 cm^3^, not surgically evacuated

Abbreviation: CT, computed tomography.

This study was performed following the ethical standards of the Ethics Committee of Human Experimentation of Fasa University of Medical Sciences with ethic number IR.FUMS.REC.1400.045 and in accordance with the 1964 Helsinki Declaration and its later amendments or comparable ethical standards.

### 2.2 Participants

Inclusion criteria for participants were as follows: (1) Moderate TBI (GCS = 9−12) at admission, (2) positive initial cranial CT scan (CT0), (3) age 18 years and older, (4) isolated blunt head injury. Exclusion criteria were: (1) Any need for a non‐neurosurgical operation, (2) depressed or compound skull fracture, (3) lacerations requiring urgent surgical intervention for repair under general anesthesia, (4) any need for neurosurgical intervention with the initial CT scan, (5) infra‐tentorial pathology, (6) mentally retarded patients, (7) patients coagulopathic (INR > 1.5, PTT > 60, or platelet count <80,000), (8) patients on anticoagulation or antiplatelet therapy (aspirin, heparin, or warfarin), (9) polytrauma causing unstable hemodynamic status.

All patients underwent a clinical assessment as well as an initial CT scan (referred to as CT 0) as part of our standard clinical protocol. Follow‐up CT scans were performed within 6 h of the initial scan for patients who had positive findings on the CT 0 scan. Unless there were signs of neurological deterioration, additional CT scans were performed every 24 h. A decrease in the GCS, a decrease in the level of consciousness, or the emergence of focal neurological deficits such as worsening headache, nausea, vomiting, changes in vision, or dizziness were considered neurological deterioration. CT scans were performed in such cases regardless of the 24 h interval to monitor the patient's condition.

After an initial examination, all patients with moderate TBI were admitted to the neuro‐ICU for primary monitoring and stabilization. All of the head CT scans were reviewed and interpreted by two neurotrauma surgeons. The radiological findings of acute intracranial pathologies such as intracranial hemorrhage, subdural hematoma, epidural hematoma, subarachnoid hemorrhage, and hemorrhagic contusion were meticulously documented. To protect the privacy of patients, all individuals were anonymized before the data analysis process. Age, gender, the cause of the trauma, the initial GCS, and clinical examination findings that indicated the need for a cranial CT, such as loss of consciousness, amnesia, vomiting, headache, somnolence, dizziness, nausea, and seizures, were all collected from the patient's hospital charts.

### 2.3 Model development

The data set was split into two partitions: the training set and the test set, based on the 70/30 split. The ML was performed using Python version 3.10.12 (Python Software Foundation) and its accompanying packages, such as Scikit‐Learn. Following the guidelines for the Scikit Learn Algorithm, we used three well‐known algorithms to determine the best predictor: Random forest (RF), support vector machine (SVM), multilayer perceptron (MLP), and logistic regression (LR).

### 2.4 RF

**Figure 1:**
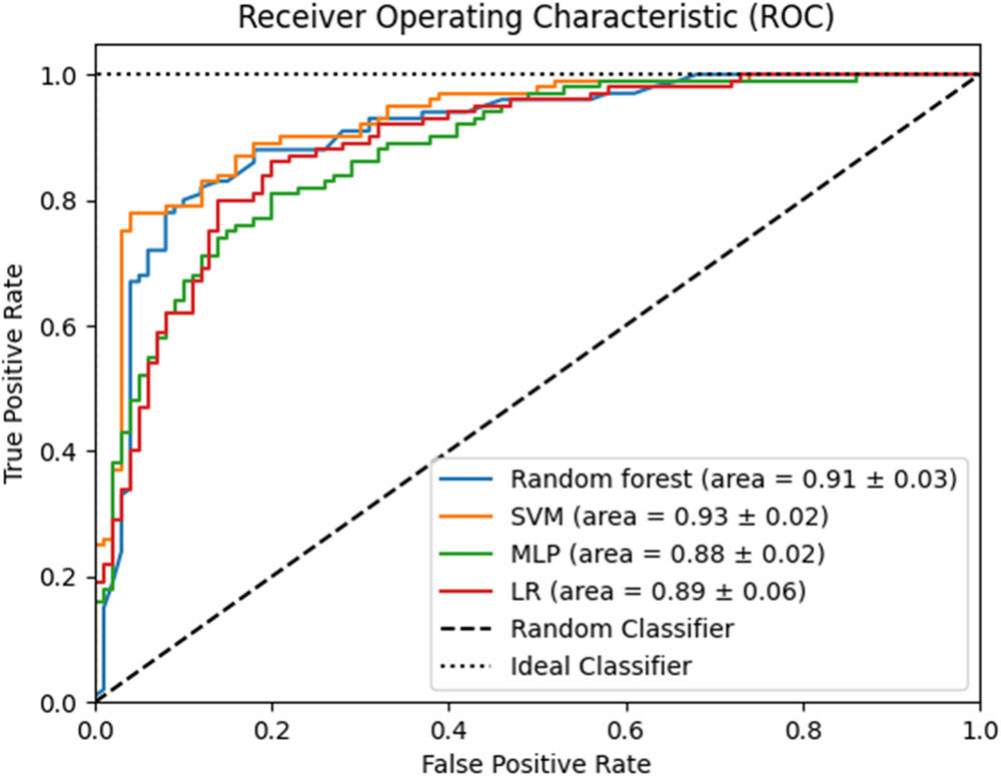
Area under receiver operating characteristic (ROC) curve (AUC) for prediction of the need for neurosurgical intervention. Values are presented as mean ± standard error. LR, logistic regression; MLP, multilayer perceptron; SVM, support vector machines.

RF is a ML technique that uses an ensemble approach to combine multiple decision trees. It builds various decision trees using different feature combinations, a process known as bootstrap aggregation. This method is especially effective at identifying relevant features within a data set. RF determines the best variable to split the data at each node in the decision tree. A subset of randomly assembled variables is used in the selection process. This method improves RF's resistance to overfitting, a common problem in ML models.^19^ To make accurate predictions, we used the RF classifier with 100 decision trees and the Gini impurity criterion in our study (Supporting Information S1: Figure 1).

### 2.5 SVM

**Figure 2:**
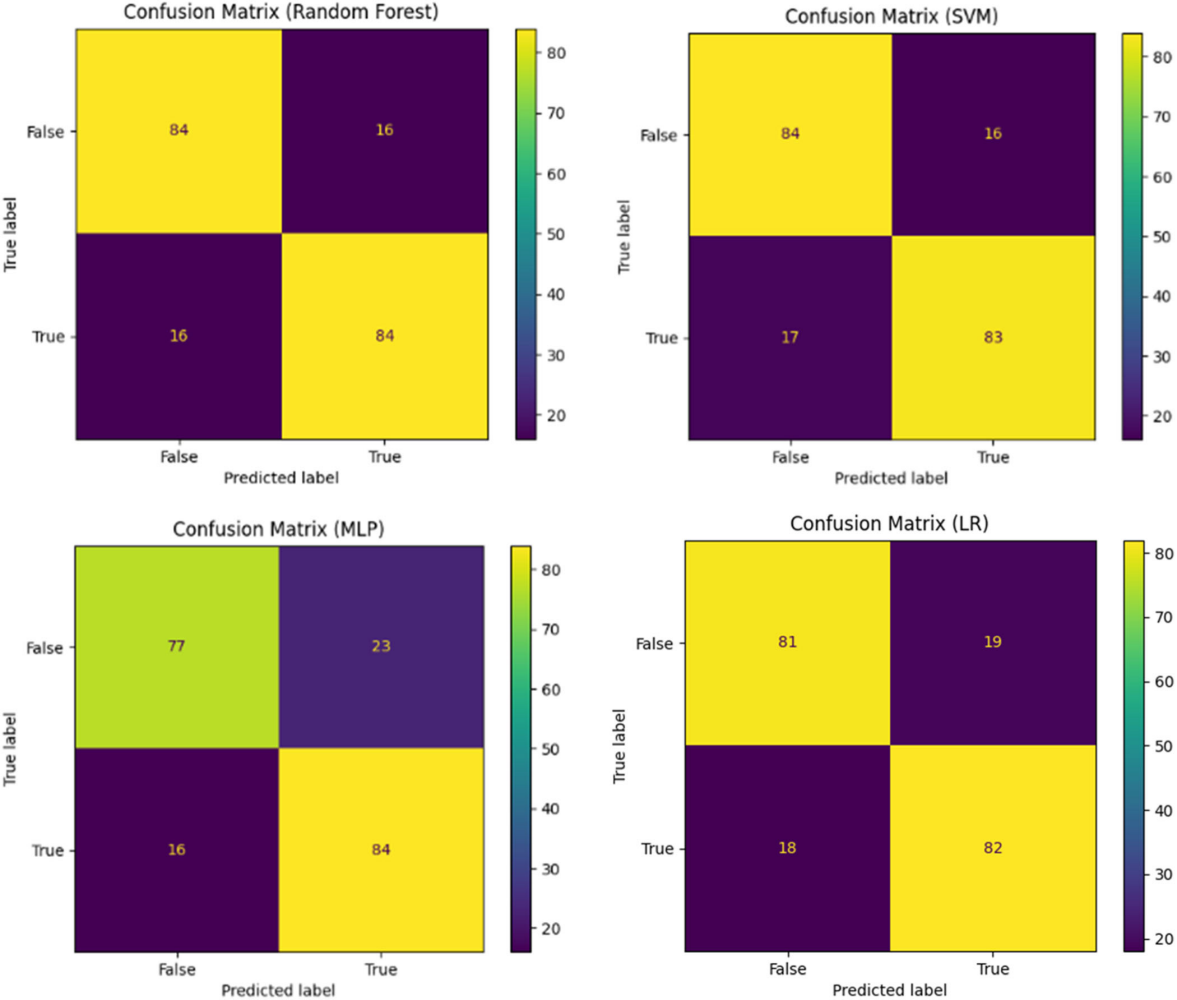
Matrices of confusion of the models: The confusion matrix describes the performance of each classification model. For example, the random forest model has a good prediction with 16 false negatives (patients that will need neurosurgical intervention but are not identified by the model) and 16 false positives (patients that will not need neurosurgical intervention but are identified by the model as needing neurosurgical intervention).

SVM is a modeling technique that predicts the value of a target variable using only attributes in the testing set. It can classify both linear and nonlinear data patterns. SVM focuses on determining a decision boundary that minimizes generalization error. SVM aims to maximize the margin between the decision boundary and the data points to accomplish this.^20^ We used the SVM classifier with a linear kernel in our study, which allows for efficient data classification based on a linear decision boundary (Supporting Information S1: Figure 2).

### 2.6 Multilayer perceptron

MLP is a type of artificial neural network made up of interconnected nodes called neurons that are arranged in multiple layers. It is a feedforward neural network, which means that information flows unidirectionally from the input layer through the hidden layers to the output layer, with no loops or cycles.^21^ In our study, we used an MLP classifier with two hidden layers of 100 neurons each. The activation function used is hyperbolic tangent (tanh), which helps determine each neuron's output. In addition, we used the “lbfgs” solver, a numerical optimization algorithm, to train and optimize the MLP (Supporting Information S1: Figure 3).

### 2.7 LR

LR is a statistical methodology predominantly employed for classification problems. The process involves determining the probability that a given data point is a member of a particular category or class. The computation in question is dependent on the utilization of a sigmoid (logistic) function, which is employed to evaluate a weighted summation of the input data. The learning process in LR entails the identification of the best values for the weights.

### 2.8 Data analysis

#### 2.8.1 Model evaluation

A confusion matrix was used to assess the performance of each method, providing insight into how well the models performed when compared to the actual values of the outcome. F1‐score, sensitivity, specificity, positive predictive value (PPV), and negative predictive value (NPV) were all calculated as test performance measures. In addition, we used the area under the curve (AUC) of the receiver operating characteristic (ROC) to evaluate the model's discriminatory ability. AUC values of 0.7 were considered acceptable discrimination, while values of 0.8 and 0.9 were considered good and excellent discrimination, respectively.^22^

We used the k‐fold cross‐validation procedure to ensure unbiased estimation and evaluation of the developed prediction models. The data set was randomly divided into k subsets, with the classifier trained on k‐1 subsets and tested on the remaining subset. This procedure was carried out k times. In our study, the samples were divided into four subgroups, three of which were used for training and one for testing. This method provided us with reliable estimates as well as an unbiased evaluation of the performance of our prediction models.

Statistical analysis was performed using IBM SPSS Statistics (version 26). Descriptive statistics, including means and standard deviations for continuous variables and frequency distributions for categorical variables, were calculated to summarize the characteristics of the study population. To assess differences in variables between the operative and nonoperative groups, the *t*‐test and *χ* ^2^ test were used.

## 3 RESULTS

A total of 200 patients meeting the inclusion criteria were included in the study of whom 100 patients (50%) required neurosurgical intervention within hospitalization. The mean age was 35.32 (19−74) and 56% (112) were male. The characteristics of the patients, both case and control, are summarized in Table 2.

**Table 2:** Patients' characteristics operative and nonoperative.

Characteristics		Operative (*N*, %)	Nonoperative	*p* Value
Age		36.18 (19−74)	34.47 (19–64)	0.231
Gender				0.557
Male		56 (56%)	56 (56%)	
Female		44 (44%)	44 (44%)	
mGCS				
mGCS0		5.30 ± 0.48	5.58 ± 0.49	0.016
mGCS6		5.12 ± 0.46	5.66 ± 0.47	0.000
Marshall				
Marshall0		2.18 ± 0.38	2.16 ± 0.37	0.401
Marshall6		2.62 ± 0.77	2.00 ± 0.23	0.000
Midline shift				
Midline shift0		3.15 ± 0.75	2.07 ± 1.05	0.001
Midline shift6		3.80 ± 1.28	2.16 ± 1.08	0.734
Hematom0				0.080
C		4	8	
S		35	26	
E		10	28	
S + E		25	30	
C + E + S		26	8	
Hematom6				0.141
C		4	8	
S		37	28	
E		10	27	
S + E		22	25	
C + E + S		27	12	
Temporal		71 (71%)	26 (26%)	0.000
Bifrontal		5 (5%)	4 (4%)	0.733

Abbreviations: C, contusions; E, epidural hematoma; mGSC, motor Glasgow coma scale; S, subdural hematoma.

### 3.1 Prediction model

To evaluate the predictive performance of each ML model, first, fourfold cross‐validation was performed on the data set. It showed that the highest F1‐score was 83%, which was achieved by both RF and SVM. The highest sensitivity was 83%, which was achieved by both RF and MLP. The highest specificity was 84%, which was achieved by both RF and SVM. The highest PPV and NPV were 84% and 85%, which both were achieved by RF (Table 3). The ROC curves of each algorithm are plotted in Figure 1, and as shown the highest AUC was 0.93, which was achieved by the SVM. The confusion matrices for the different models are presented in Figure 2.

**Table 3:** Performance of the machine learning‐based prediction developed for predicting the need for neurosurgical intervention in patients with moderate traumatic brain injury.

Models	F1‐score	Sensitivity	Specificity	PPV	NPV
LR	0.81 ± 0.05	0.82 ± 0.07	0.81 ± 0.06	0.81 ± 0.05	0.82 ± 0.04
RF	0.83 ± 0.07	0.83 ± 0.10	0.84 ± 0.09	0.84 ± 0.08	0.83 ± 0.06
SVM	0.83 ± 0.07	0.82 ± 0.07	0.84 ± 0.07	0.83 ± 0.09	0.83 ± 0.04
MLP	0.80 ± 0.06	0.83 ± 0.04	0.78 ± 0.05	0.78 ± 0.08	0.83 ± 0.02

*Note*: Values are presented as mean ± standard error.

Abbreviations: LR, logistic regression; MLP, multilayer perceptron; NPV, negative predictive value; PPV, positive predictive value; RF, random forest; SVM, support vector machines.

## 4 DISCUSSION

### 4.1 Summary of the results and background

Our study aimed to develop the first ML‐based model that could predict the need for neurosurgical intervention in patients with moderate TBI. The findings demonstrated that ML models could accurately predict the need for neurosurgical intervention in moderate TBI patients with high sensitivity and specificity. These findings imply that ML‐based early prediction models have the potential to improve timely triage and optimize resource utilization in the management of patients with moderate TBI.

TBI is a major public health concern, resulting in numerous emergency department visits. In addition, it is a leading cause of morbidity and mortality in young adults.^23^ Therefore, it is regarded as a major societal issue with far‐reaching socioeconomic consequences.^1^, ^24^ Expert and timely management is essential to avoiding long‐term complications and mortality. This is challenging, particularly in limited‐resource settings. There is a need to create risk stratification tools for TBI to tackle this problem.

While there are guidelines for treating patients with mild and severe TBI, there is a notable lack of clear guidance for treating patients with moderate TBI.^25^, ^26^, ^27^ The literature focuses primarily on mild or severe TBI, with little attention given to moderate head injuries. A significant number of patients with moderate TBI who are initially conscious and communicative may deteriorate or even die.^28^, ^29^ Therefore, a comprehensive understanding and management of moderate TBI is crucial to improving neurological outcomes.^3^ In light of this, we focused on treating patients with moderate TBI, as it represents a complex and demanding domain in neurotrauma.

PHI is a complication of TBI that can occur after the initial brain injury.^6^ It is a severe and devastating complication. Previous research has shown that patients who experience PHI have a higher risk of clinical deterioration and poorer outcomes than those who do not experience PHI.^7^ PHI prevalence in TBI patients has been reported to range from 8% to 67%.^6^, ^30^

Predicting PHI is one of the most frequently cited reasons for routine follow‐up head CT scans. According to studies, 20% of intracranial findings may appear deteriorated on follow‐up head CT scans, but they do not always necessitate treatment changes or surgery.^31^ Routine follow‐up head CTs alone do not ensure that the patient's condition will not worsen in the future.^32^

Several attempts have been made to assess the value and timing of repeated brain CT scans.^33^, ^34^ In a retrospective study, Trevisi et al.^33^ investigated the value of repeated brain CT scans in patients with mild TBI and positive initial CT findings. They analyzed 222 patients and found that patients with mild TBI and posttraumatic intracranial lesions at the initial CT scan had minimal benefit from repeated follow‐up CT scans. However, they suggested that a second CT scan within 48 h may still be necessary for cases involving subdural hematoma or subarachnoid hemorrhage to avoid excessive scanning.

Joseph et al.^35^, ^36^ conducted a study aiming to reduce the frequency of routine follow‐up brain CT scans in patients with TBI. They developed the Brain Injury Guidelines (BIG) protocol, which integrated patient history, neurological examination, and findings from the initial head CT scan to determine the appropriate course of action for each individual. The implementation of the BIG protocol resulted in a 29% decrease in routine follow‐up CT scans, a 20% decrease in neurosurgery consultations, and no change in mortality rates. Furthermore, there were no changes in neurosurgical interventions or readmission rates after the protocol implementation.^37^

According to another study, follow‐up CT scans did not lead to neurosurgical intervention in 99% of patients.^38^ There is, however, the issue of the low event rate, which may have limited the ability of the sample to identify these patients accurately. A discriminating approach is needed that prioritizes clinical evaluation and monitoring rather than routine follow‐up CTs.

Several clinical and demographic variables have been identified as predictors of PHI.^39^ We trained our models based on some of the most critical features measured upon admission and 6 h after admission. These features include age, gender, motor GCS 0, motor GCS 6, Marshall 0, and Marshall 6. And CT scan data such as 0‐ and 6‐h hematomas, midline shift, temporal lesions, and bifrontal contusions.

Age, gender, and motor GCS importance on PHI has been widely discussed in the literature.^40^, ^41^ Another key factor is the type and location of the hematomas, as well as the contusions.^42^ Cerebral contusions are distinguished by their dynamic and expanding nature, particularly in the first few hours following trauma. These lesions progress to about 45% on average.^43^, ^44^ Contusions are treated the same as intracranial hematomas in our study, as long as the volume is less than 50 cc. If the contusion volume exceeds 10 cc, it is considered a major pathology. We divided the patients into five groups based on their major pathology: sole contusions, sole subdural hematoma, sole epidural hematoma, subdural and epidural, and subdural or epidural with contusion.

The presence of bifrontal contusions in patients was one of the variables considered in our study. Bifrontal contusions can swell and cause brain displacement, resulting in sudden deterioration as the brain stem descends into the posterior fossa. This can result in respiratory arrest, coma, and autonomic dysfunction.^45^ Neurosurgeons face difficulties managing patients with significant bifrontal contusions.^46^, ^47^ The onset of deterioration varies, nonetheless, brain edema typically peaks around the fifth to tenth day and then resolves. As a result, close observation in an ICU for up to 2 weeks is advised, as well as follow‐up CT scans every 2−3 days to monitor the progression of the lesions.^29^, ^46^, ^47^

Finally, we also included initial and 6 h Marshall scores as our variables. The Marshall classification score, first described in 1992, is a commonly used measurement derived from CT scans.^48^ It has shown predictive abilities for patient outcomes in TBI cases. The significance of the Marshall score lies in its capacity to provide valuable prognostic information, assisting clinicians in making informed decisions during TBI management. While the Marshall score is widely utilized, studies have identified limitations, such as its inability to differentiate between epidural and subdural hematomas. In addition, it has limited predictive value for intracranial mass lesions.^49^, ^50^, ^51^ However, when used with other variables, the Marshall score can still contribute to outcome prediction.

### 4.2 The ML algorithms

Studies have shown that through ML, it is possible to detect small clinical markers and trends that may remain undetected during routine clinical observation. This provides valuable insight into the resuscitation of TBI patients.^52^, ^53^ In various medical situations, including TBI, ML has shown promise in predicting patient outcomes and determining the likelihood of deterioration or the need for intervention.^54^

Our models achieved balanced prediction by balancing specificity and sensitivity, yielding fair predictions for both groups. Furthermore, our models' AUC values were comparable to other algorithms. It is important to note that the performance of a prediction model is heavily dependent on the data used to train and test it. When data varies or is unavailable, it becomes difficult to compare multiple models. The results of our study indicate that a reliable predictive model for the need for neurosurgical intervention could be created by combining a set of simple features with interpretable ML‐based models. The SVM model, particularly, outperformed the RF and MLP models. MLPs are deep learning models with multiple neuron layers. They have a high capacity to learn intricate patterns from data but may require larger data sets to generalize effectively. In our case, the limited sample size might have constrained the MLP's ability to discover the underlying patterns in the data. Also, neural networks, including MLPs, perform highly dependent on fine‐tuning hyperparameters such as layers, neurons per layer, and learning rates. In larger data sets, there may be more room for extensive hyperparameter search and optimization. SVM's higher performance could be attributed to its ability to identify the hyperplane that maximizes the margin between classes, allowing it to establish clear decision boundaries. In a medical classification task, such as predicting neurosurgical intervention, having well‐defined boundaries between classes is critical, and SVM excels at this. The relatively lower performance of MLP in our study could be attributed to the combination of a modest sample size and the complexity of the model. While RF and SVM are known for their ability to perform well with smaller data sets and fewer assumptions, neural networks like MLPs often excel with larger and more diverse data. These promising results show that ML‐based models have the potential to predict outcomes for TBI patients. A decision‐support tool such as this could assist clinicians in diagnosis, treatment optimization, and improving clinical outcomes.

Another advantage of our models is that we focus on a limited number of readily accessible variables in daily clinical practice. This differs from other ML‐based models that use more variables. Our models are easy and simple to use in real‐world healthcare settings, making them ideal for decision‐making.

Recently, several ML algorithms have been developed to help decision‐making for TBI patients based on baseline data.

Moyer et al.^54^ aimed to develop an ML model to predict the need for emergency neurosurgery within 24 h after moderate to severe TBI. They trained and tested various models on a cohort of 2159 patients. The best‐performing model achieved an AUC of 0.81 and identified several predictive variables, including initial GCS, high blood pressure, and low heart rate. Another study by Adil et al.^55^ focused on TBI in low‐ and middle‐income countries and the importance of effective triage and decision‐making regarding neurosurgery. ML models predict patient outcomes with and without surgery. The models were validated using cross‐validation, and various predictors were considered, including clinical variables and surgical intervention. The models achieved high predictive performance, with ROC values ranging from 0.83 to 0.88. The models highlighted the importance of variables such as GCS and receiving surgery in promoting good outcomes. A recent study by Tunthanathip et al.^56^ compared the predictive performance of ML algorithms and a nomogram in identifying intracranial injury following cranial CT in pediatric patients with TBI. Data from 964 patients were used to train and validate the models. The RF algorithm provided the highest performance, with an AUC of 0.80. These studies suggest that ML algorithms have the potential to support physicians in reducing the overuse of head CT scans and lowering treatment costs in TBI patients.

### 4.3 Implications of the findings and future directions

To the best of our knowledge, we present the highest‐performing predictive models for TBI patients based on data from the first hours These models provide novel and personalized predictions about the need for surgery. Our study protocol is simple and easy to implement in any emergency department. Moderate TBI patients can be assessed and classified efficiently with readily available triage criteria. Our prediction models’ outcomes serve as early warning notifications, allowing healthcare providers to plan future care based on predicted risk. By combining patient‐specific demographics and clinical presentation to generate individualized predictions, this tool advances personalized medicine beyond the average treatment effects derived from randomized clinical trials. Clinicians in the trauma resuscitation unit can enter patient data to appropriately triage patients for nonoperative management or CT scans followed by potential surgery, validating the models' efficacy and practical application in real‐world clinical settings. This method maximizes using limited medical resources while lowering unnecessary imaging costs for patients and their families. Expanding data sets, developing mobile applications for low‐resource settings, conducting field tests with local clinicians, and identifying implementation barriers through surveys are the next steps in improving ML‐based models for medical prediction.

### 4.4 Limitations

One of our study's major limitations is the small sample size, which may impact the accuracy of the ML models. Larger sample sizes are typically required to achieve more precise predictions in ML models. Collaborations between multiple centers could be established in the future to improve ML‐based models' performance.

Our ML model was based on CT findings and data from the first 6 h. However, future models should aim to incorporate only the initial CT findings, allowing for faster use of prediction results and optimization of treatment strategies. Also, certain parameters, such as lab data and the mechanism of injury, were not taken into account in our study.

Furthermore, since our study used samples obtained retrospectively from one institution, it is possible for the ML models proposed to not be applicable to other institutions due to the risk of bias. For more reliable data, a prospective cohort study is recommended.

## 5 CONCLUSION

Our study shows that ML‐based models are effective decision‐making tools for accurately predicting the need for neurosurgery following moderate TBIs. It is critical to integrate these models into existing workflows and conduct prospective evaluations to fully understand their efficacy in real‐world situations and influence clinical decision‐making. Finally, these models have the potential to be decision‐support tools for evaluating early interventions in TBI patients and improving patient care and outcomes.

## AUTHOR CONTRIBUTIONS

**Adrina Habibzadeh**: Conceptualization; data curation; methodology; visualization; writing—original draft. **Hossein Ali Khalili**: Conceptualization; supervision; writing—review and editing. **Sepehr Khademolhosseini**: Formal analysis; visualization; writing—original draft. **Amin Kouhpayeh**: Conceptualization; investigation. **Amin Niakan**: Supervision; writing—review and editing. **Mohammad Ali Asadi**: Formal analysis; methodology. **Hadis Ghasemi**: Resources; validation. **Reza Tabrizi**: Conceptualization; data curation; investigation; project administration; resources; writing—review and editing. **Reza Taheri**: Conceptualization; investigation; methodology; project administration; resources; validation; writing—review and editing.

## CONFLICT OF INTEREST STATEMENT

The authors declare no conflict of interest.

## ETHICS STATEMENT

This study was performed after obtaining the ethics approval of Fasa University of Medical Sciences with the number IR.FUMS.REC.1400.045.

## TRANSPARENCY STATEMENT

The lead author Reza Taheri affirms that this manuscript is an honest, accurate, and transparent account of the study being reported; that no important aspects of the study have been omitted; and that any discrepancies from the study as planned (and, if relevant, registered) have been explained.

## Supporting information

Supporting information.

## Data Availability

The data sets used and/or analyzed during the current study are available from the corresponding author upon reasonable request.
