# Supplementary material for: Machine learning‐based models to predict the need for neurosurgical intervention after moderate traumatic brain injury
Source: Health Sci Rep. 2023 Oct 29;6(11):e1666. doi: 10.1002/hsr2.1666 (PMC10613807; doi:10.1002/hsr2.1666)
Supplement: Supplementary file 1 — Supporting information. [file HSR2-6-e1666-s001.docx]

Supplementary information


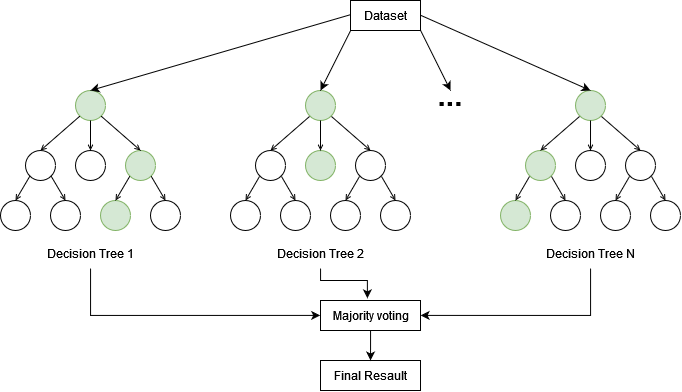


Figure 1. Random Forest


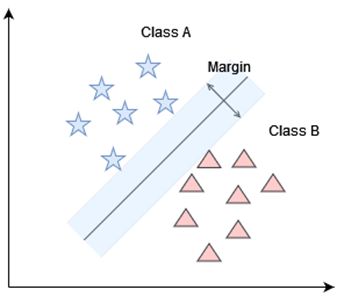


Figure 2. Support vector machine


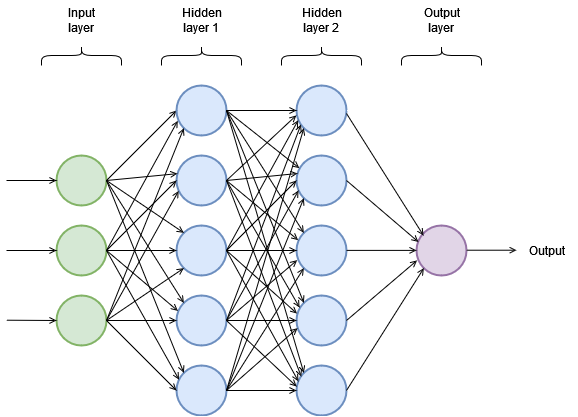


Figure 3. Multi-layer Perceptron
